# Neuritin-overexpressing transgenic mice demonstrate enhanced neuroregeneration capacity and improved spatial learning and memory recovery after ischemia-reperfusion injury

**DOI:** 10.18632/aging.202318

**Published:** 2020-12-15

**Authors:** Kexing Wan, Fuxiu Mao, Qiongqiong Li, Limin Wang, Zhiguo Wei, Ping Wang, Xinhua Liao, Mengsi Xu, Jin Huang, Zemin Pan, Chengtan Wang, Jian Luo, Rui Gao, Shangquan Gan

**Affiliations:** 1 Department of Biochemistry, College of Medicine, Shihezi University, Shihezi 832000, Xinjiang, China; 2 State Key Laboratory of Sheep Genetic Improvement and Healthy Production, Xinjiang Academy of Agricultural and Reclamation Sciences, Shihezi 832000, Xinjiang, China; 3 College of Animal Science and Technology, Henan University of Science and Technology, Luoyang 471023, Henan, China; 4 College of Animal Food and Bioengineering, Henan University of Science and Technology, Luoyang 471023, Henan, China; 5 First Affiliated Hospital, Medical College, Shihezi University, Shihezi 832000, Xinjiang, China

**Keywords:** transient global ischemia, transgenic mice, neuritin, nerve injury and repair

## Abstract

Acute ischemia-reperfusion (IR)-induced brain injury is further exacerbated by a series of slower secondary pathogenic events, including delayed apoptosis due to neurotrophic factor deficiency. Neuritin, a neurotrophic factor regulating nervous system development and plasticity, is a potential therapeutic target for treatment of IR injury. In this study, Neuritin-overexpressing transgenic (Tg) mice were produced by pronuclear injection and offspring with high overexpression used to generate a line with stable inheritance for testing the neuroprotective capacity of Neuritin against transient global ischemia (TGI). Compared to wild-type mice, transgenic mice demonstrated reduced degradation of the DNA repair factor poly [ADP-ribose] polymerase 1 (PARP 1) in the hippocampus, indicating decreased hippocampal apoptosis rate, and a greater number of surviving hippocampal neurons during the first week post-TGI. In addition, Tg mice showed increased expression of the regeneration markers NF-200, synaptophysin, and GAP-43, and improved recovery of spatial learning and memory. Our findings exhibited that the window of opportunity of neural recovery in Neuritin transgenic mice group had a tendency to move ahead after TGI, which indicated that Neuritin can be used as a potential new therapeutic strategy for improving the outcome of cerebral ischemia injury.

## INTRODUCTION

Disruption of neurovascular perfusion (stroke) frequently results in permanent loss of neural function and concomitant motor and/or cognitive disability. About 30 million people worldwide are disabled by stroke every year, and another 6 million die from stroke. Stroke has surpassed heart disease to become the second leading cause of death globally. More than 795,000 people in the United States suffer a stoke each year, of which about 610,000 are new (first stroke) cases [1]. In general, current treatments are effective only in the earliest post-stroke phase. Moreover, stroke-related disability places a major burden on healthcare resources, so preventative treatments or more effective post-stroke therapies are needed, particularly for aging populations.

Stroke can be divided into two types, ischemic and hemorrhagic, of which 87% are of the global ischemic type. Numerous animal studies have shown that as little as 6 minutes of ischemia is sufficient to cause delayed neuronal death in the CA1 region of the hippocampus. However, most neurons die 3–7 days after the ischemic event, providing a therapeutic window for treatment [2–4]. The initial reduction in energy stores and the DNA fragmentation induced by disruption of ionic balance among intracellular, extracellular, and subcellular compartments initiate a cascade of pathogenic signaling events culminating in delayed apoptosis [5–7]. Neurons are highly differentiated terminal cells, and so have limited repair and regenerative capacity. Further, the microenvironment of the central nervous system (CNS) is unfavorable to axonal and neurite regeneration. Therefore, current treatment effects are limited and prognosis is generally poor. The primary goals of experimental stroke treatments are to rescue salvageable neurons by blocking pathogenic events associated with apoptosis (e.g., inflammation and oxidative stress) and provide a microenvironment more conducive to neuronal repair and restoration of functional connectivity [8].

Numerous studies have shown that neurotrophic factors delivered to damaged nerve endings can improve axonal regeneration by inducing expression of reparative molecules [9–11]. Indeed, genetic manipulation of neurotrophic factor expression, such as nerve growth factor (NGF) or glial-derived neurotrophic factor (GDNF) overexpression, have shown success in experimental models of clinical diseases including stroke [12, 13]. *Neuritin* is a neurotrophin first cloned in 1993 from a cDNA library of rat hippocampal dentate gyrus by Nedvi and initially named plasticity-related candidate gene 15 (CPG15). It was subsequently renamed *Neuritin* in 1997 based on findings that CPG15 promoted the rapid growth of neurites [14–17]. *Neuritin* is primarily expressed in cortex, hippocampus, lateral geniculate body, trigeminal ganglion, and olfactory bulb of developing embryos. In adulthood, *Neuritin* is expressed mainly in regions with high neuroplastic potential such as hippocampus, olfactory bulb, and the cerebellar Purkinje cell layer [18–20]. Numerous studies conducted with cell culture models have demonstrated that Neuritin promotes neurite growth [16], axonal branching and synaptic development [19], formation of synaptic circuits [21], and neuronal migration [22], while inhibiting neuronal apoptosis [23]. These findings suggest Neuritin as a potential candidate molecule for neuronal rescue and repair. The neuroprotective efficacy of Neuritin has also been extended to multiple animal models. For instance, Neuritin promoted the recovery of damaged hippocampal neurons in a transient global ischemia (TGI) model [24, 25], enhanced nerve cell survival, neurite regeneration, and recovery of motor function in a spinal cord injury (SCI) model [26, 27], and preserved retinal structure and function in the mouse optic nerve crush injury (ONI) model [28–30]. Wang et al reported that recombinant human Neuritin stimulated nerve regeneration and restored motor function in a rat model of sciatic nerve injury [31], while Liu et al reported that Neuritin reduced neuronal apoptosis induced by traumatic brain injury [32] and Zhang et al found that Neuritin protected against early brain injury in a subarachnoid hemorrhage model [33]. In addition to acute nerve damage models, Neuritin has been reported to mitigate neural and behavioral symptoms in several psychiatric disease models such as the chronic unpredictable stress model of depression [34–41]. In addition, Neuritin can promote the differentiation of non-neural cells into neural lineage cells, promote the growth of neurites from damaged neurons, and prolong neuronal survive in culture [21–22, 42–43].

Although Neuritin has demonstrated neuroprotective efficacy in various acute and chronic neurological disease models, most of these were simple *in vitro* cellular models in which the insult and physiological environment could be precisely controlled. In addition, these studies used the mature exogenous protein obtained by genetic engineering without the glycosylphosphatidylinositol (GPI) anchor domain, so there was no specific subcellular targeting effect. Moreover, the exogenous recombinant Neuritin polypeptide fragment is poorly penetrant across the blood-brain barrier (BBB) and so must be delivered by intracerebroventricular (ICV) administration, which may cause secondary injury and is impractical as a potential clinical treatment strategy. Also, the exogenous recombinant Neuritin peptide fragment is not maintained at an effective therapeutic concentration after injection as it is easily degraded by the immune response, resulting in limited promotion of repair and recovery of neurological function. Therefore, the neuroprotective efficacy of endogenous full-length Neuritin remains unclear. To overcome many of these shortcomings, we established a transgenic mouse model stably overexpressing exogenous Neuritin including the GPI, and compared neural damage and recovery to wild-type mice following transient global ischemia-reperfusion.

## RESULTS

### Construction of recombinant plasmid pcDNA3.1(+)-neuritin

The map of the recombinant pcDNA3.1(+)-neuritin vector is presented schematically in Supplementary Figure 1A. We first successfully amplified the complete rat *Neuritin* coding region with 3’ *NheI* and 5’ *XhoI* restriction sites. The amplified targeted DNA fragment and pcDNA3.1(+) plasmid were double digested by *NheI* and *XhoI*, yielding the expected fragment sizes of 429 bp and 5,344 bp, respectively, after purification (Supplementary Figure 1B, 1C). Positive transformants were identified by PCR and plasmids extracted for further identification by restriction enzyme digestion (Supplementary Figure 1D). The accuracy of the recombinant Neuritin plasmid was then confirmed by sequencing (Supplementary Figure 1E), which indicated 100% identity with the reference rat *Neuritin* nucleotide sequence in GenBank (NM_053346).

### Preparation of transgenic mice and screening for Nrn1 overexpression

### Pronuclear injection for the production of transgenic mice

The full-length pcDNA3.1(+)-neuritin transgenic recombinant plasmid (5773 bp) was double digested with *StuI* and *BglII* endonucleases, and the band carrying the target transgenic DNA fragment (2.38 kb) was recovered (Supplementary Figure 2A, 2B), purified, and diluted to 3 ng/μL for injection.

More than 1,200 zygotes were collected from superovulated females mated with males (Supplementary Figure 2C). In total, 711 cleaned zygotes were successfully injected with target DNA (Supplementary Figure 2D) and then re-implanted into 26 foster mothers (Supplementary Figure 2E). Finally, a total of 45 F0 offspring were produced. Positive transgenic mice were identified by PCR detection of the CMV sequence and one copy of the Nrn1 transgene in genomic DNA from tail samples (Supplementary Figure 2F). To ensure integration of the complete target DNA fragment, only simultaneous appearance of the expected 1081-bp band of the CMV promoter (Supplementary Figure 2F, left) and the 508-bp band of the complete Nrn1 gene (Supplementary Figure 2F, right) was considered transgene-positive. There were no differences in appearance, behavior, or feeding between positive transgenic mice (Nrn1-Tg) and WT littermates.

### Screening and propagation of transgenic mice overexpressing neuritin

**Figure 1:**
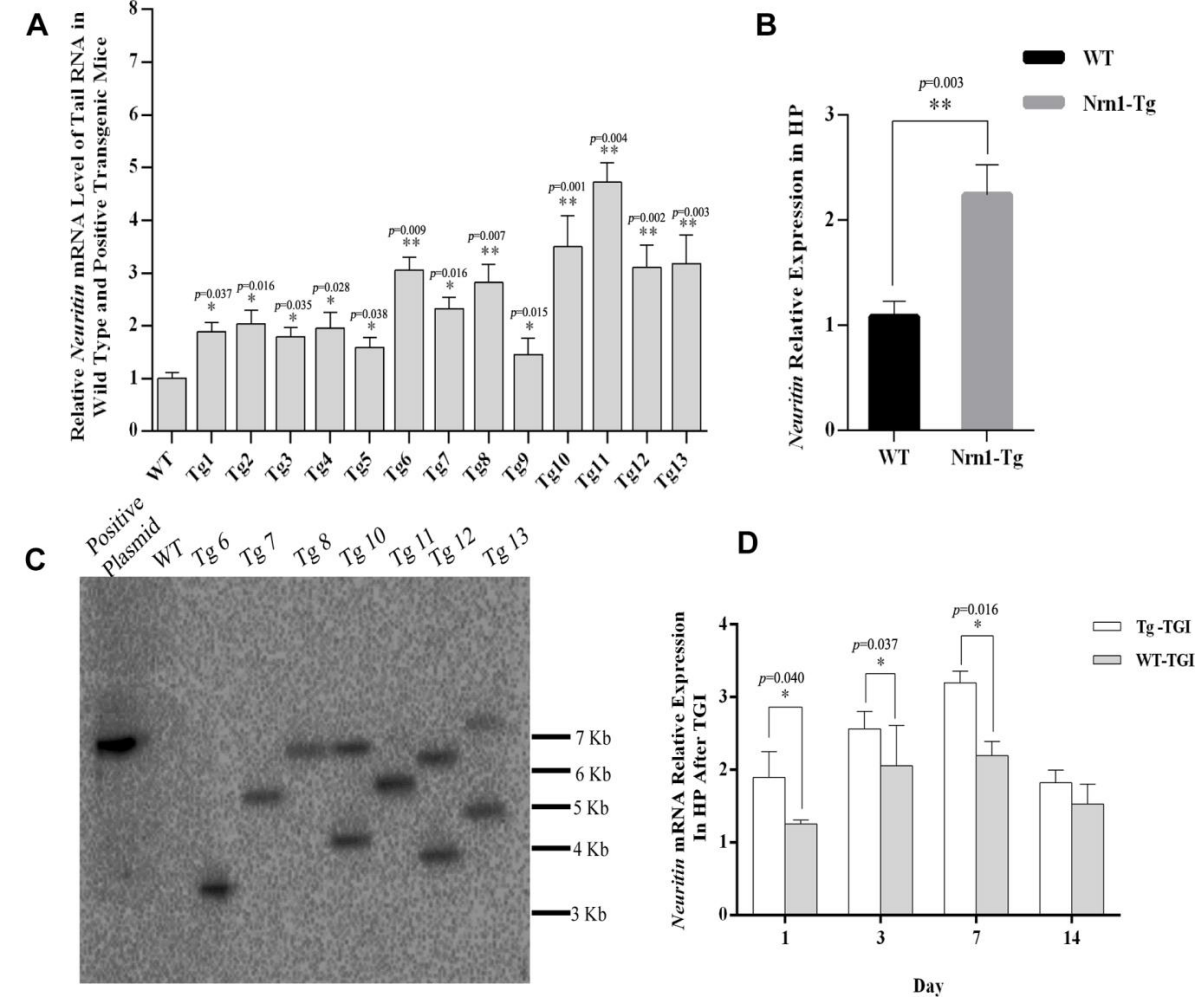
**Generating neuritin-overexpressed transgenic mouse line and constructing transient global ischemia model of transgenic mice.** (**A**) Neuritin mRNA level in tail tissue of wild-type (WT) and transgenic (Nrn1-Tg) mice. (**B**) Neuritin mRNA expression level in hippocampus (HP) of the screened Nrn1-Tg and WT groups. (**C**) Southern blotting analysis to evaluate the number of integration site in transgenic mice identified by qRT-PCR. Lane 1: pcDNA3.1-neuritin plasmid. Lane 2: genomic DNA of wild type mouse. Lanes 3-9: genomic DNA of transgenic mice with the transgene expression. (**D**) Neuritin mRNA level in hippocampus of Nrn1-Tg and WT mice on day 1, 3, 7, and 14 post-TGI. GAPDH was used as the internal control. Data expressed as mean ± SEM and compared by Student’s *t*-test (*p*<0.05 considered statistically significant). *p< 0.05, **p< 0.01 versus WT, n=3 mice per group with triplicate biological replicates.

**Figure 2:**
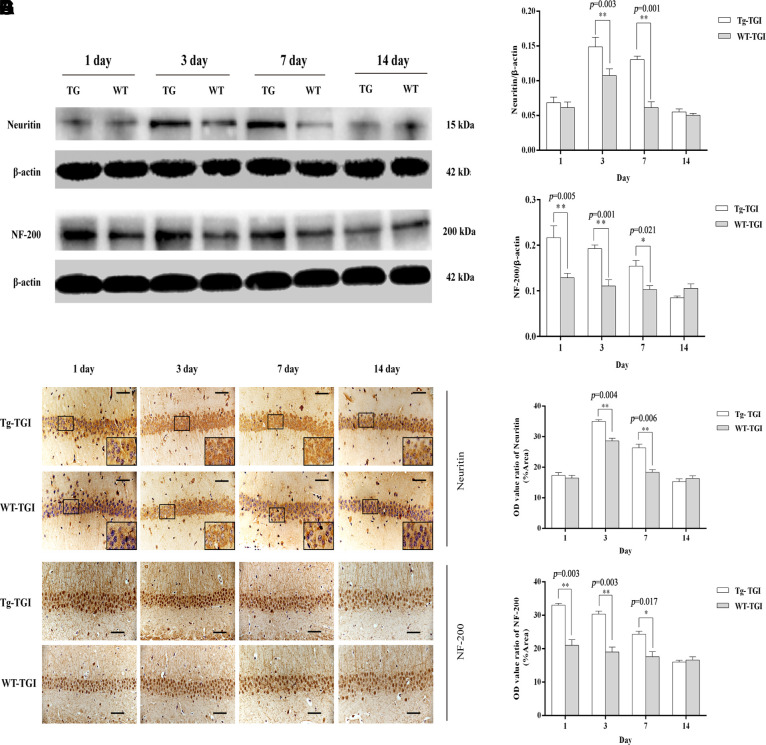
**Protective efficacy of neuritin overexpression revealed by upregulation of neurite markers.** (**A**) Western blots of Neuritin and NF-200 expression level at the indicated time points after TGI. β-actin was used as the gel loading control. Tg-TGI, neuritin transgenic mice subjected to TGI; WT-TGI, WT mice subjected to TGI. (**B**, **C**) Quantitative analysis of neuritin and NF-200 expression shown in (**A**) respectively. (**D**) Immunohistochemical analysis of Neuritin expression in the hippocampal CA1 region at the indicated time points after TGI. Small black rectangular frame/large black rectangular frame=1/4. (**E**) Quantitative analysis of Neuritin expression data (OD value ratio) shown in (**D**). (**F**) Immunohistochemical analysis of NF-200 expression in the hippocampal CA1 region at the indicated time points after TGI. (**G**) Quantitative analysis of NF-200 expression data (OD value ratio) shown in (**F**). Data expressed as mean ± S.E.M. (n = 6). scale bar=50 μm, *p < 0.05, **p < 0.01 and ***p < 0.001 vs. WT-TGI group.

To properly assess the effect of exogenous Neuritin on neural injury and recovery following TGI, it is critical that transgenic offspring exhibit consistent and physiologically significant gene overexpression. Therefore, we preliminarily identified transgenic offspring by detecting *Neuritin* mRNA expression in tail clips by qRT-PCR, the expression level of *Neuritin* mRNA in Nrn1-Tg mice was higher than that in WTs (Figure 1A). Seven founders with a 2–5-fold increase in transgene expression (compared with the controls) were further chosen to evaluate the transgenic integration site, of which four single-site-integrated transgenic mice Tg6, 7, 8 and 11 were identified by southern blot (Figure 1C). The presence of a single copy of a transgene with a single integration site is ideal for producing a pure transgenic line. In this study, one Tg7 mouse with an approximate one-fold increase in hippocampal neuritin expression was selected for generating the transgenic mouse line (Figure 1B). Transgenic mice were then further screened for stable inheritance and expression to ensure that Neuritin protein levels were at least one-fold higher than in WTs for further TGI experiments (Figure 2A). We also confirmed that elevated expression relative to WTs was maintained on day 1, 3, and 7 post-TGI in animal model (Figure 1D).

### Neuritin-overexpressing Tg mice enhanced neuroregeneration capactity after TGI

**Figure 3:**
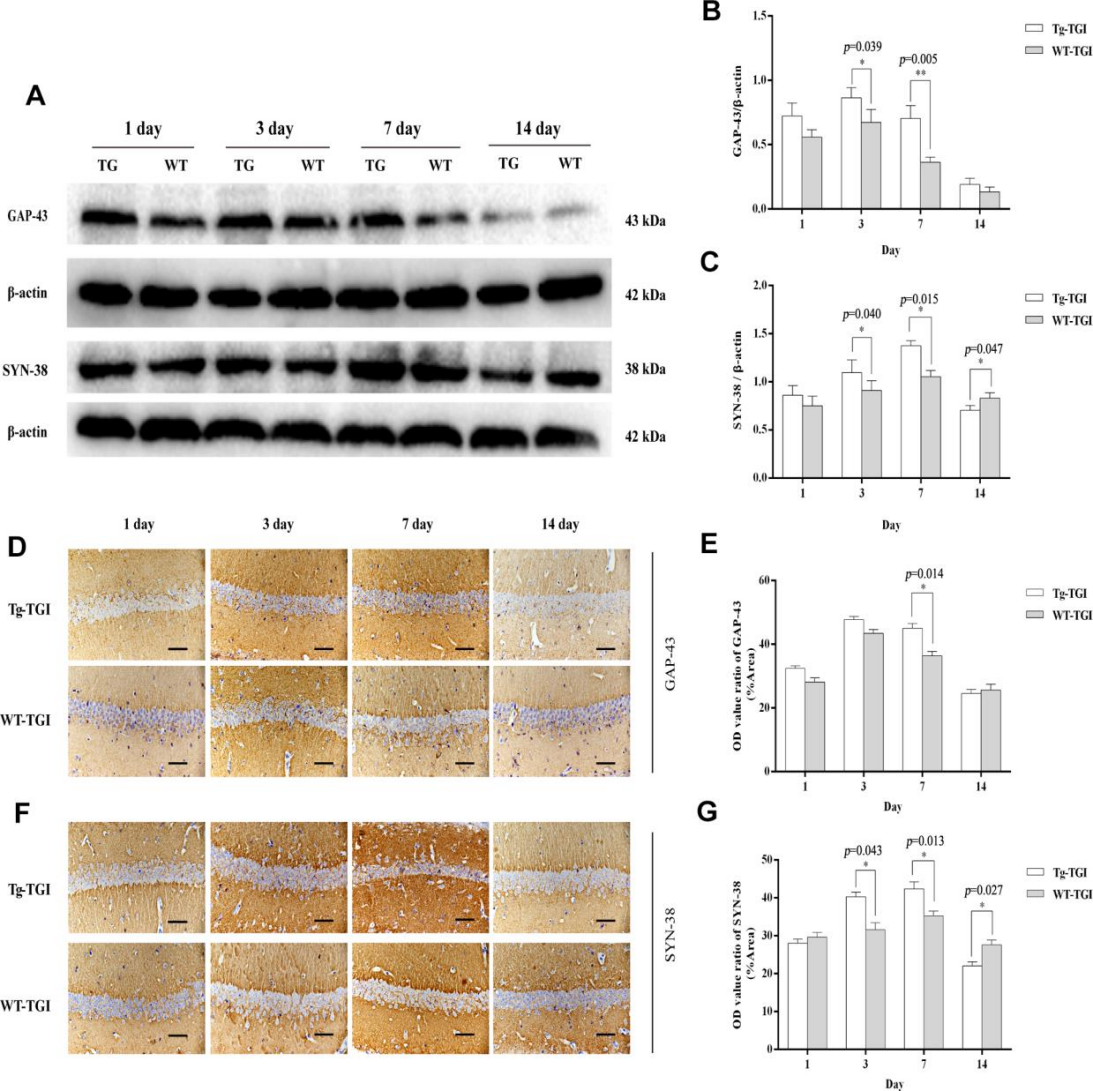
**Protective efficacy of neuritin overexpression on cerebral ischemia−reperfusion injury revealed by upregulation of synaptic markers.** (**A**) Western blots of GAP-43 and synaptophysin (SYN-38) expression at the indicated times after transient global ischemia (TGI). β-actin was used as the gel loading control. (**B**, **C**) Quantitative analysis of the Western blot results for GAP-43 and SYN-38 shown in (**A**), respectively. Protein bands were quantified by optical density (OD) measurements. (**D**, **F**) Protein expression levels of GAP-43, SYN-38, respectively, in hippocampal CA1 by immunohistochemistry. (**E**) Quantitative analysis of the immunohistochemistry results for GAP-43 shown in (**D**). (**G**) Quantitative analysis of the immunohistochemistry results for SYN-38 shown in (**F**). Tg-TGI: neuritin-overexpressing transgenic mice subjected to TGI; WT-TGI: wild-type mice subjected to TGI. Six randomly chosen brain sections from three mice were used for statistical analysis. Data are expressed as mean ± S.E.M. n = 6 mice per group, scale bar=50 μm, *p < 0.05, **p < 0.01 versus WT group.

To examine whether Neuritin overexpression can enhance synaptic reconstruction and neurite regeneration, expression levels of the synaptic markers GAP-43 and SYN-38 and the neurite marker NF-200 in hippocampus/hippocampal CA1 were measured by western blotting and immunohistochemistry with optical density (OD) quantification respectively. In the present study, expression levels of GAP-43 and SYN-38 proteins first increased and then decreased in both Nrn1-Tg and WT mice following TGI, but peak expression levels on days 3 and 7 post-TGI were significantly higher in the Tg-TGI group, as detected by western blot analysis and quantitative analysis (Figure 3A–3C). NF-200 expression was significantly higher in Nrn1-Tg than in WT mice at each time point from 1 to 7 days post-injury, as detected by western blot and quantitative analysis (Figure 2A–2C). We further performed immunohistochemistry assay in hippocampal CA1 region (the most sensitive region response to hypoxia-ischemic brain injury) (Supplementary Figure 3), similar expression trends (GAP-43, NF-200 and SYN-38) were observed when compared to the results detected by western blot and quantitative analysis respectively (Figure 3D–3G and Figure 2F–2G). And *Neuritin* also shows a similar expression pattern to that of above markers (Figure 2A, 2B, 2D, 2E). These results suggest stronger activation of reparative processes.

### Neuritin-overexpression in Tg-TGI mice can alleviate nerve damage and suppress neuronal apoptosis

**Figure 4:**
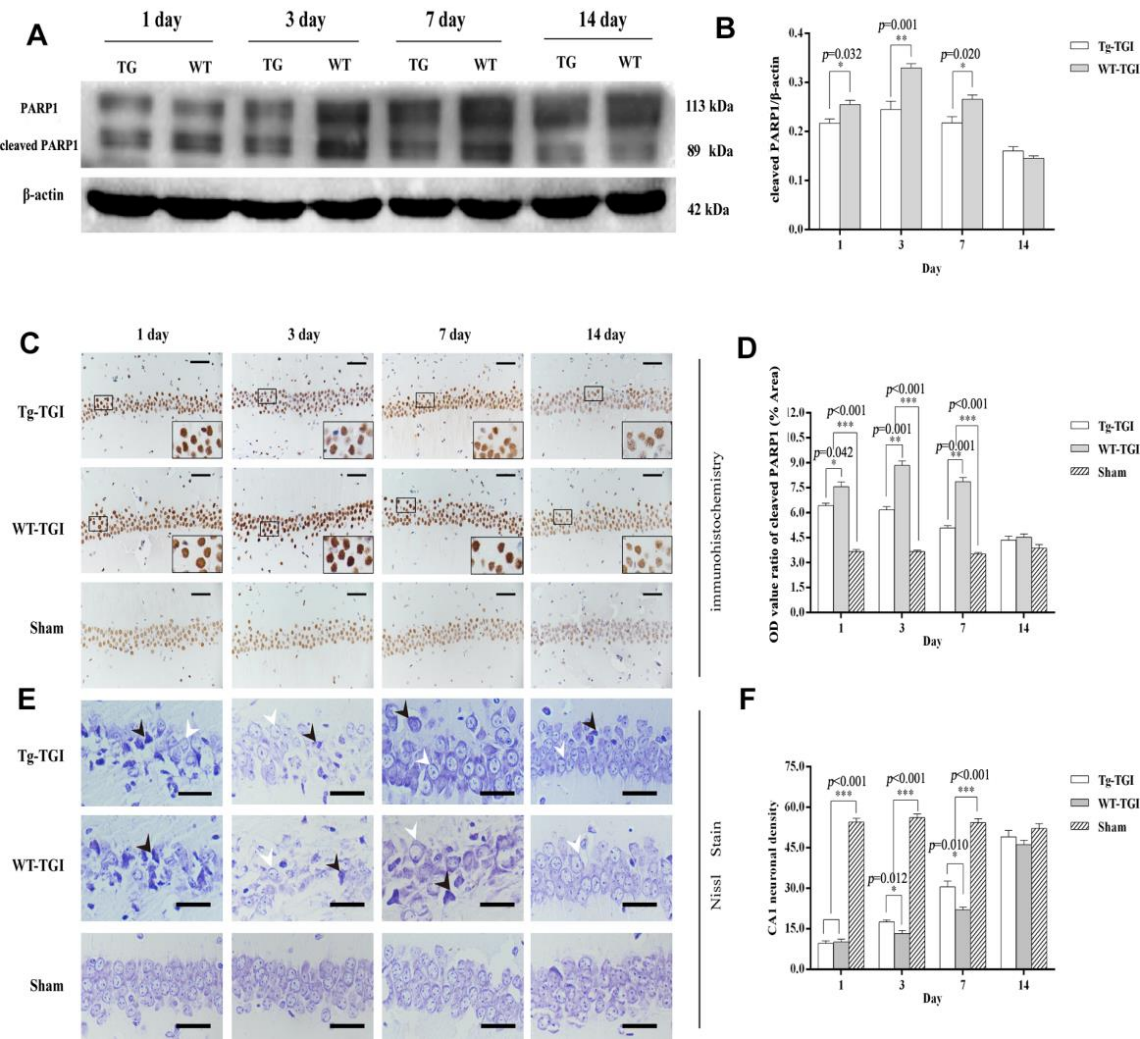
**Suppression of TGI-induced apoptosis by neuritin overexpression.** (**A**) Western blots of PARP 1 expression level as an index of apoptosis at the indicated times after TGI. β-actin was used as the gel loading control. Tg-TGI, neuritin transgenic mice subjected to TGI; WT-TGI, WT mice subjected to TGI. (**B**) Quantitative analysis of cleaved PARP 1 expression data (OD value ratio) shown in (**A**). (**C**) PARP 1 protein expression levels in hippocampal CA1 of sham and TGI groups at different time points revealed by immunohistochemistry (scale bar=50 μm). Small black rectangular frame/large black rectangular frame=1/4. (**D**) Quantitative analysis of expression (OD value ratio) shown in (**C**). (**E**) Representative photographs of Nissl-stained hippocampal CA1 at the indicated time points after TGI (scale bar=25 μm). Black arrows indicated the damaged neurons, white arrows indicated the normal neurons. (**F**) Quantitative analysis of Nissl staining from (**E**). Cell counts (number of neurons per 200 μm length) from six sections of left and right hippocampus were averaged for each animal. Data expressed as mean ± S.E.M. n = 6 mice per experimental group, *p < 0.05, **p < 0.01 and ***p < 0.001 versus the sham and WT groups, respectively.

Cleaved PARP 1 protein expression is a key index of apoptosis, and hippocampal expression levels were significantly higher in WT than Nrn1-Tg mice on days 1−7 after TGI. Similar to markers of neural repair, cleaved PARP 1 protein expression did not differ between groups on day 14 post-TGI (Figure 4A, 4B). Thus, Neuritin overexpression appeared to suppress the secondary wave of neuronal apoptosis in hippocampus within the first week following TGI.

Numerous studies have shown that a brief (5-min) transient global cerebral ischemia episode can induce selective loss of neurons in hippocampal area CA1. In this study, we further studied PARP 1 protein levels by immunohistochemistry in CA1. Expression levels were significantly higher in both groups on days 1−14 post-TGI compared to corresponding sham groups, but were significantly higher in the WT group than the Tg-TGI group on days 3 and 7 (Figure 4C, 4D). The results of Nissl staining further showed that the pyramidal cells in hippocampal CA1 regions of TGI model groups were loose and disordered when compared with those in the sham group. Normal neurons had relatively larger cell bodies, abundant cytoplasm, and one or two big round nuclei (white arrow). In contrast, the injured neurons showed markedly shrunken cell bodies, condensed nuclei, dark cytoplasm, and many empty vacuoles (black arrow; Figure 4E). Nissl staining and cell counting also revealed that the number of CA1 neurons was markedly reduced in both WT and Tg-TGI CA1 post-TGI (Figure 4E). Furthermore, compared with the WT-TGI group, the number of normal neurons in hippocampal CA1 was significantly higher in the Tg-TGI group on days 3 and 7. On day 14, there were no differences in neuron counts among groups. Thus, Nrn1 overexpression accelerated the early recovery in CA1 neuron numbers following TGI (Figure 4F).

### Spatial learning and memory improvement in Tg mice after TGI

**Figure 5:**
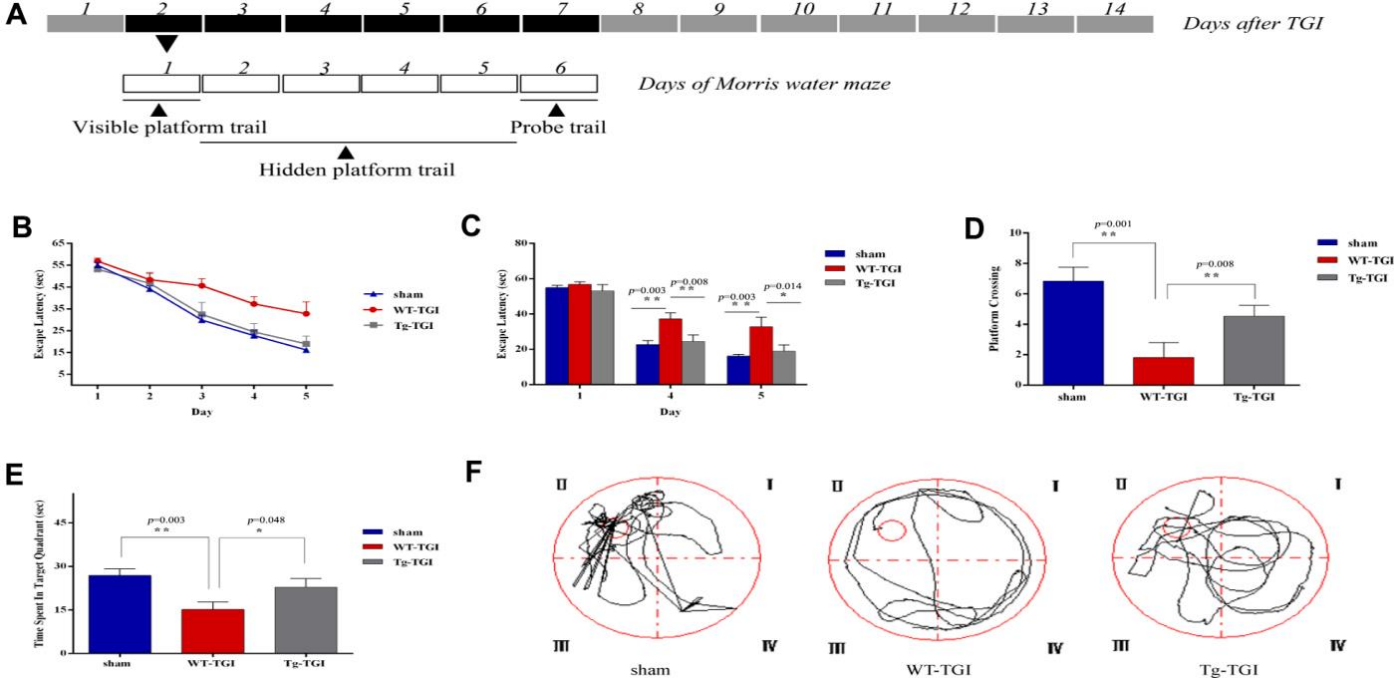
**Neuritin overexpression improves recovery of spatial learning and memory following transient global ischemia.** (**A**) Morris water maze (MWM) schedule and experimental design. Dark boxes represent days after TGI, of which, black boxes represent critical stage of nerve repair. White boxes represent days of Morris water maze. MWM experiments started from on day 2 and ended on day 7 of TGI. (**B**) Escape latencies on the visible platform trials (day 1) and hidden platform trials (days 2–5). There were no group differences in escape latencies among groups in visible platform trials. Latencies decreased progressively during hidden platform trials, but faster in the Tg-TGI group than the WT-TGI group. (**C**) Bar graph showing no significant group differences in escape latencies on visible platform trials (day 1) but significantly shorter latencies for Tg-TGI and sham groups on the final two invisible platform trial days (days 4 and 5). (**D**−**F**) Results of the probe trial for spatial memory (day 6). (**D**) Tg-TGI mice made significantly more crossings of the original platform position than WT-TGI mice. (**E**) Tg-TGI mice spent significantly more time in the original platform quadrant than WT-TGI mice. (**F**) Example swim paths demonstrating distinct search strategies by Tg-TGI and WT-TGI mice (targeted vs. random). Data are presented as mean ± SEM (Sham: n=12, WT-TGI: n=12, Tg-TGI: n=11). Legend: *p<0.05; **p<0.01 versus WT-TGI and sham, respectively, by one-way analysis of variance (ANOVA) followed by LSD tests for pair-wise comparisons.

According to our histochemical and western blotting results, the first week post-TGI is a critical period for neuronal apoptotic death and recovery. Further, indices of degeneration were lower and indices of recovery higher in the hippocampus of Tg-TGI mice compared to WT-TGI mice during this period, collectively suggesting faster recovery of hippocampal function. To directly investigate hippocampal function post-TGI, we compared performance in the MWM test during this critical period (Figure 5A). On trials in which the hidden platform was marked with a visible cue (day 1), there were no group differences in escape latency (Figure 5B, 5C), indicating that Tg-TGI and WT-TGI mice exhibited similar motor and visual capabilities as well as motivation. In hidden platform trials assessing spatial learning, the progressive reduction in escape latency during days 2−5 was markedly accelerated in the Tg-TGI group compared to the WT-TGI group, and such that latencies were significantly lower on days 4 and 5. In fact, the rate of learning as indicated by latency did not differ between the Tg-TGI group and the sham group (Figure 5B, 5C). During the probe trial, Tg-TGI mice also made a significantly greater number of platform crossings (Figure 5D) and spent a significantly greater proportion of the trial time in the target (former platform) quadrant (Figure 5E). Again, these indices were nearly equivalent to those of sham mice, indicating that Nrn1 overexpression preserved spatial memory. Typical swimming paths (Figure 5F) also revealed a more targeted search strategy by Tg-TGI mice, while WT-TGI mice tended to swim randomly among the quadrants.

## DISCUSSION

Cerebral ischemia-reperfusion injury involves both early necrotic neuronal death due to energy failure and swelling and a later phase of delayed apoptosis due to oxidative damage on reperfusion, release of inflammatory cytokines, and loss of neurotrophic factors among other processes. In the late stage of reperfusion, neural damage and apoptosis result in reduced secretion of neurotrophic factors that normally serve to maintain the survival and functional integrity of neurons, thereby further aggravating IR injury. Timely supplementation of neurotrophic factors in animal models can improve repair following cerebral ischemia and slow the progression of neurodegenerative diseases [44–47]. Overexpression of Neuritin was also found to improve cognitive function in Alzheimer's disease models by increasing the density of dendritic spines and restoring neurite structure [37, 48]. Sharma et al [31] found that recombinant Neuritin protein can maintain retinal ganglion cells and promote the regeneration of axons after optic nerve injury. Our previous studies showed that recombinant Neuritin can promote neurite outgrowth from PC12 cells and prolong the survival of chick embryo dorsal root ganglia [49, 50]. In addition, exogenous Neuritin dose-dependently enhanced recovery from acute sciatic nerve and spinal cord injury [26, 51]. Collectively, these studies indicated that Neuritin not only prevented degeneration via apoptosis but also promoted neurite outgrowth, branch formation, synaptic plasticity, and synaptogenesis for functional recovery. Thus, enhanced Neuritin signaling appeared to be a potential therapeutic strategy for acute and chronic neurodegenerative diseases. Further, there was a temporal correlation between *Neuritin* upregulation and the recovery of neurological function after cerebral IR injury. However, due to the limited upregulation of endogenous Neuritin functional restoration is usually incomplete. Moreover, due to rapid degradation, direct application *in vivo* is of little benefit [27].

Mature Neuritin contains a GPI domain that targets the protein to specific regions of the cell membrane and is necessary for optimal receptor-mediated transmembrane signal transduction [17]. It is difficult to obtain a full-length protein containing the GPI anchor domain using genetic engineering techniques, while exogenous recombinant Neuritin protein is rapidly degraded. Moreover, the recombinant Neuritin protein does not localize without the GPI site and so cannot reach sufficient concentrations within the damaged area. Therefore, we established *Neuritin* transgenic mice in which overexpression was strongly driven by the CMV promoter.

The choice of promoter is a key factor driving the expression of the target gene, and CMV is a constitutive promoter that induces strong overexpression of exogenous genes in almost all mammalian cells without tissue specificity. It has been widely used to construct highly efficient eukaryotic expression vectors. Asselbergs et al. compared expression efficiency among the CMV promoter, SV40 early promoter, an adenovirus late promoter, and found highest expression using the CMV promoter [52]. Xiang et al also compared CMV to SV40 early promoters for expression of rabies virus glycoprotein *in vitro* and found strongest expression using the CMV promoter [53]. The neuroprotective efficacy of Neuritin in this study may stem from both inclusion of the GPI anchor and consistently high expression driven by the CMV promoter.

We successfully screened for a highly overexpressing transgenic founder mouse prior to mass breeding. After mating with WT mice, about half of the progeny stably inherited *Neuritin* overexpression, but only mice with Neuritin gene transcript overexpression more than 2-fold higher than WT as confirmed by qRT-PCR were selected to produce the experimental population. Furthermore, we confirmed that there were no nonspecific effects on development or behavior and that overexpression was maintained following IR injury. Neuritin overexpression reduced the early loss of hippocampal CA1 neurons, upregulated proteins indicative of neurite regeneration and synaptogenesis, decreased expression of an apoptosis marker, and enhanced spatial learning and memory compared to WTs following TGI.

Neuritin overexpression in hippocampus significantly inhibited PARP 1 protein degradation. PARP 1 is activated by damaged DNA fragments, and once activated protects DNA from hydrolysis by nucleases and directs damaged DNA to bind repair enzymes [54]. When DNA is severely damaged, however, PARP 1 is split into two fragments, P89 and P24, by caspase-3 and caspase-7, which inhibits DNA repair and ultimately induces apoptosis [55, 56]. Nissl staining demonstrated that Neuritin overexpression can reduce neuronal loss and accelerate functional recovery after cerebral IR. Previous studies have shown that Neuritin can inhibit neuronal apoptosis by inhibiting caspase-3 activation [21, 25]. Thus, Neuritin overexpression may block caspase-3 activation after cerebral IR injury, thereby inhibiting PARP 1 protein degradation and subsequent DNA damage, reducing neuronal apoptosis, rescuing damaged neurons, and maintaining functional capacity (as evidenced by MWM performance).

Regeneration of damaged axons and reconstruction of synaptic connections to achieve functional restoration is the ultimate goal of stroke therapy [57]. Axonal buds on surviving neurons are the basis for network reconstruction. Growth-associated protein 43 (GAP-43) is an important biomarker for assessing axonal regeneration. It is highly expressed during development and in axonal growth cones after nerve injury, where it regulates the reassembly of the neural cytoskeleton for axonal growth and path-finding [58, 59]. Neurofilament 200 (NF-200) is also essential for axon growth, maintenance of axon morphology, and axoplasmic transport [60, 61]. Synaptophysin (SYN-38) is a presynaptic marker involved in the transport of synaptic vesicles, vesicular release of neurotransmitters, synaptic vesicle recycling, and synaptogenesis. Expression level can be used to predict changes in the number or density of synapses, and hippocampal expression is strongly associated with spatial cognition [62, 63]. Neuritin overexpression significantly increased NF-200 and GAP-43 levels in CA1 neurons during the early critical period of axonal regeneration after cerebral IR injury. We assume that GAP-43 and NF-200 upregulation in turn promoted axonal sprouting and dendrite branching, resulting in improved hippocampal function as evidenced by MWM test performance. At the same time, upregulation of SYN-38 expression would be conducive to establishing new connections among dendrites and axons, thereby restoring the functional circuitry required for hippocampal-dependent spatial memory.

Neuritin is not only enriched in the CNS, but also in various cancers such as astrocytoma, glioma, Kaposi's sarcoma, and invasive breast cancer, where it regulates tumor cell proliferation and apoptosis [21, 64–66]. Thus, overexpression of Neuritin may have potentially deleterious effects inside and outside the CNS. While Nrn1-Tg mice did not exhibit obvious phenotypic abnormalities compared to WTs, it is still desirable to upregulate Neuritin specifically in response to CNS injury. This will be a major focus of our future studies.

This study demonstrated that upregulation of Neuritin expression in transgenic mice can reduce neuronal damage, inhibit neuronal apoptosis, increase expression of reparative molecules (NF-200, GAP-43, and SYN-38) in the highly IR injury-prone hippocampus, and mitigate deficits in hippocampal-dependent spatial learning and memory. Functional impairment of the hippocampus may be related to apoptotic death of neurons in dentate gyrus and CA1/CA3 areas [67, 68]. These results indicated that a sufficient local concentration of Neuritin can provide a favorable microenvironment for maintaining neuronal survival and neurite outgrowth, reduce degeneration and apoptosis of injured neurons, restore tissue metabolic capacity, and accelerate the synthesis of structural and functional proteins required for axonal regeneration and synaptogenesis, thereby shortening the time required for functional recovery. These findings may provide new strategies for the development of drugs to treat ischemic stroke.

## MATERIALS AND METHODS

### Animals

Adult C57BL/6 mice weighing ~25 g (range 22–28 g) were supplied by the Experimental Animal Center of Xinjiang Medical University. All animal procedures were conducted in strict accordance with the Ethics Guidelines of the First Affiliated Hospital of Shihezi University Medical College (Xinjiang Province, China).

### Vector construction and pronuclear injection

### PcDNA3.1(+)-Nrn1 plasmid construction

The eukaryotic pcDNA3.1(+) vector (Clontech, Mountain View, CA, USA) carrying the constitutive cytomegalovirus (CMV) promoter was used as the transgene vector. The entire Nrn1 coding sequences with terminal *NheI* and *XhoI* restriction sites was cloned from rat RSC96 cell cDNA via PCR amplification using the primers Nrn1-NheI-F, 5'-CTA GCT AGC ATG GGA CTT AAG TTG AAC GGC-3' and Nrn1-Xho1-R, 5'-CCG CTC GAG TCA GAA GGA AAA GCC AGG TCG C-3' (the underlined letters indicate *Nhe I* and *Xho I* restriction enzyme sites, respectively). The PCR thermocycle conditions were as follows: 94° C for 5 min followed by 35 cycles of 94° C for 30 s, 58° C for 30 s, and 72° C for 30 s. The pcDNA3.1(+)-Nrn1 plasmid was constructed by inserting the Nrn1 coding sequences between the NheI and XhoI restriction sites of the pcDNA3.1(+) vector. The pcDNA3.1(+)-Nrn1 plasmid was then digested with *BglII* and *StuI* (Fermentas, MBI, USA), a linearized DNA fragment containing the CMV promoter and Nrn1 was recovered by removing macromolecular contaminants, and the purified 2.3-kb DNA fragment was used for pronuclear microinjection.

### Production of transgenic mice by pronuclear injection

Four- to 5-week-old C57 females were intraperitoneally injected with 10 IU of pregnant mare’s serum gonadotropin (PMSG, Sigma, MO, USA), followed 48 hours later by human chorionic gonadotropin (HCG, Sigma, MO, USA) injection. Females were then housed overnight with adult males and the next day checked for the presence of a vaginal plug. Inseminated mice were sacrificed by cervical dislocation and the fertilized eggs obtained from the fallopian tubes. The collected eggs were treated with hyaluronidase to remove granulosa cells, and then subjected to male pronuclear microinjection. Fifteen to twenty surviving zygotes were implanted into the oviduct of each pseudopregnant C57 foster mother according to established procedures [69].

### Identification of neuritin transgenic mice and evaluation of transgene expression

A 0.5-cm section of the tail-tip was cut, placed in a 1.5-mL centrifuge tube containing 0.5 ml digestion buffer (100 mM Tris HCl pH 8.5, 5 mM EDTA, 0.2% (w/v) SDS, 20 mM NaCl, and protease K (5 μL × 20 mg/mL)), and incubated overnight at 55° C with shaking. Genomic DNA was isolated using the standard phenol/chlorine extraction method. To verify genomic integration of the entire fragment including the CMV promoter and Nrn1 coding region, samples were analyzed by PCR using two sets of primers, Nrn1-TgF1: 5'-GCC AGT ATC TGC TCC CTG-3', Nrn1-TgR1: 5'-CCT GGC AAT CCG TAA GAG-3', Nrn1-Tg F2: 5'-GTG GAT AGC GGT TTG ACT C-3', and Nrn1-TgR2: 5'-CCT GGC AAT CCG TAA GAG-3', and the following thermocycle: 35 cycles of 94° C for 30 s; 60° C for 30 s; 72° C for 1 min.

For selection of transgenic mice with high Nrn1 expression, total RNA was isolated from the tail tips of both positive transgene mice and wild-types (WTs) using TRIzol reagent (Invitrogen, Carlsbad, CA, USA) and subjected to quantitative real-time PCR (qRT-PCR). Only transgenic mice (Nrn1-Tg) with one-fold higher Nrn1 gene expression relative to WTs were used as F1 offspring for breeding.

### Transient global ischemia (TGI) model construction

Female and male Nrn1-Tg and WT mice at 3−4-months-old and 20−28 g were used for TGI experiments. Seventy-two mice were divided into sham-operated control (n=24), Tg-TGI (n=24) and WT-TGI (n=24) groups. The TGI model was established as previously described [25]. Briefly, after induction of anesthesia with 4% chloral hydrate (0.01 mL/g body wt), an incision was made in the median neck. Animals in the TGI groups were then subjected to bilateral suture occlusion of the common carotid arteries (CCAs) for 15 min. Sham-operated animals were treated identically except for bilateral CCA occlusion. Some of these mice were sacrificed 1, 3, 7, or 14 days after surgery for western blotting and qRT-PCR. Tissue from the cerebral cortex and hippocampus were removed within 5 min and immediately flash-frozen in liquid nitrogen.

### Morris water maze

The Morris water maze (MWM) test is a robust and reliable method for evaluating spatial learning and memory capacity, and test performance is strongly related to hippocampal function. The MWM test was conducted according to pervious reports [70, 71] with some modifications.

### Apparatus

The Morris water maze test was performed in a white circular tank (diameter = 122 cm, height = 51 cm) filled with clean water to a depth of approximately 36 cm and maintained at approximately 23° C. A movable circular transparent Perspex platform (diameter = 10 cm, height= 34.5 cm) was placed in the tank with the top submerged about 1.5 cm below the water surface. The circular tank was divided into four quadrants (I, II, III, and IV) and the platform was positioned an equal distance from the center and edges in the middle of quadrant II. Animals were required to find the platform under both visible and invisible conditions as described below. Performance was recorded using a ceiling-mounted camera linked to the Ethovision XT7 program (Noldus, Wageningen, The Netherlands).

### Spatial reference memory acquisition

On the first day of the memory acquisition (training) phase, a flag was placed on the submerged platform and mice were simply required to find and mount it (visible platform version) to assess vision, swimming ability, and motivation. At the beginning of each trial, a mouse was lifted by the base of the tail and gently placed into the water facing the wall. Each trial began at one of four pseudo-randomly chosen start positions with the platform maintained in a fixed position. On days 2−6, each mouse performed four consecutive trials per day without the platform marker (invisible platform test). The mice were allowed a maximum of 60 s to find the platform. If the mouse did not arrive at the platform within 60 s, it was placed on the platform and allowed to remain there for 30 s. If the mouse found the platform within 60 s, it was allowed to stay for the inter-trial interval of 15 s.

### Probe trial test

To assess spatial memory for hidden platform location, mice performed a probe test 24 h after the final spatial acquisition trial. The platform was removed and the mouse was placed at a novel start position 180° from the original platform position. The mouse was allowed to swim freely for 60 s and memory of the (former) platform location was assessed by the number of platform location crossovers and time spent in the target quadrant (II).

### Southern blotting

To analyze integration sites of transgenic mice (Tg 6-8 and 10-13) with the transgene expression identified by qRT-PCR, genomic DNA was isolated by using phenol-chloroform extraction method, pcDNA-3.1-neuritin plasmid DNA and WT genomic DNA were taken as the positive and negative controls, respectively. Genomic DNA (100 ug) and plasmid DNA (10 pg) were digested with *EcoRI* for 12 h in a 37° C water bath and then separated on a 1% (w/v) agarose gel. The agarose gel was transferred onto a positively charged Hybond-N+ nylon membrane (GE Healthcare, Uppsala, Sweden) with a Trans-Blot SD system (Bio-Rad) followed by UV-crosslinking. The probe was labeled using a PCR digoxygenin probe synthesis kit (Roche Applied Science, Indianapolis, IN, USA), the forward and reverse primers were as follows: F, 5’-GCC AGT ATC TGC TCC CTG-3’(forward) and R, 5’-CCG CTC GAG TCA GAA GGA AAA GCC AGG TCG C-3’ (reverse). Hybridization and Immunologic process was conducted following the manufacturer’s instructions of DIG-high prime DNA labeling and detection starter kit II (Roche Applied Science).

### Western blotting

Frozen cortex and hippocampal tissues were homogenized in RIPA lysis buffer containing proteinase inhibitor for 30 min on ice and centrifuged at 12,000 × *g* for 10 min. The protein concentration in the supernatant was determined by BCA assay, and 20 μg total supernatant protein per gel lane was separated by 12% SDS-PAGE and transferred to 0.45 μm nitrocellulose membranes (Biosharp Life Sciences, China).

After blocking, membranes were probed with the following primary antibodies: mouse monoclonal anti-Nrn1 (1:200, Abcam, Cambridge, MA, USA), rabbit monoclonal anti-synaptophysin (SYN-38, 1:10,000, Abcam), rabbit monoclonal anti-GAP-43 (1:10,000, Abcam), mouse monoclonal anti-hypophosphorylated neurofilament H (1:1,000, Abcam, Cambridge, MA, USA), rabbit monoclonal anti-PARP 1 (1:1,000, Abcam, Cambridge, MA, USA), and mouse monoclonal β-actin (1:2,000, ZSGB-BIO, Beijing, China) as the gel loading control. Immunoreactivity was detected using horseradish peroxidase (HRP)-conjugated goat anti-mouse or anti-rabbit antibodies (1:5,000 dilution, Zhongshan Jinqiao Biotechnology Co., Beijing, China). Protein bands were visualized by an enhanced chemiluminescence system and band intensity was quantified using ImageJ software (NIH, Bethesda, MD, USA).

### Quantitative RT-PCR

Total RNA was isolated from tail clips of transgenic and WT mice using TRIzol reagent (Invitrogen) according to the manufacturer’s instructions. RNA concentration and purity were assessed using the NanoDrop 2000 spectrophotometer (Thermo Scientific, Scotts Valley, CA, USA). First-stand cDNAs were synthesized from 500 ng of total RNA using the Prime Script^TM^ RT Master Mix (Perfect Real Time; Takara, Dalian, China) according to the supplier’s protocol. Real-time PCR reactions were then conducted using 2×SYBR^®^ Premix Ex Taq™ II (Takara, Dalian) and the LightCycler^®^ 96 Real-Time PCR System (Roche, Basel, Switzerland). Glyceraldehyde-3-phosphate dehydrogenase (GAPDH) was used as the reference gene. Target genes were amplified in a 15 μL reaction volume using the following thermocycle conditions: 94° C for 2 min and then 40 cycles of 94° C for 30 s, 60° C for 30 s, and 68° C for 30 s. All qRT-PCR reactions were performed with three biological replicates. Expression levels were calculated relative to GAPDH expression using the 2^-ΔΔCT^ method. The forward and reverse primer sets were as follows:

Nrn1-F: 5'- GCA TGG CCA ACT ACC C-3'; Nrn1-R: 5'-CCT TCC TGG CAA TCC GT- 3'; GAPDH F:5'-TGA CGT GCC GCC TGG AGA AA-3'; GAPDH R, 5'-AGT GTA GCC CAA GAT GCC CTT CAG-3'

### Histology and immunohistochemistry

Paraffin-embedded brain sections were cut at 4-μm thickness, deparaffinized in xylene, and rehydrated in graded ethanol. Sections were then subjected to antigen retrieval by heating in 0.1 M sodium citrate buffer (pH 6.0) for 20 min. Endogenous peroxidase activity was quenched by incubation in 3% H_2_O_2_ for 15 min. The sections were then immunostained overnight at 4° C with one of the following primary antibodies; anti-neuritin (1:100), anti-PARP 1 (1:1,000), anti-neurofilament (NF)-200 (1:200), anti-GAP-43 (1:1,000), or anti-SYN-38 (1:800). After washing three times with PBS (5 min/wash), the sections were incubated with goat anti-rabbit or anti-mouse secondary antibody at 37° C for 1 h, washed three times in PBS (5 min/wash), and counterstained with hematoxylin. Negative control sections were incubated with PBS instead of primary antibody. Immunolabeling was visualized by DAB reagents (Beijing Zhong-shan Biotechnology Co. Ltd., Beijing, China). Representative images were acquired from non-overlapping fields of hippocampus using a light microscope (CX21, Nikong, Japan) and immunohistochemical staining was quantified as optical density (OD) using ImageJ 1.46a.

### Nissl staining

Nissl staining was used for histological examination and assessment of neuronal cell loss. Sections of 4-μm thickness were washed twice for 15 min in 0.01 M PBS and incubated with Nissl Staining Solution (Beyotime Institute of Biotechnology, Nanjing, China) for 30 min at 37° C. Sections were then washed with double distilled water, dehydrated in graded ethanol (70%, 95%, and 100%), made transparent with xylene, and mounted on cover slips using mounting medium. To quantify the surviving neurons in the hippocampal CA1 region, clear and intact neural cells with Nissl bodies uniformly distributed around the nuclei in the hippocampal CA1 regions were counted under a light microscope by two investigators blinded to the experimental design. Normal hippocampal neurons in the medial CA1 cell layer were counted in 200 μm intervals in six sections per animal under a light microscope. Cell counts from the left and right hippocampus in each of the six sections were averaged to provide a single value (number of neurons per 200 μm length) for each animal [72].

### Statistical analysis

All data are presented as mean ± SEM. Group means were compared by one-way analysis of variance (ANOVA) followed by LSD tests using the general linear model of the Statistical Package for Social Sciences (SPSS, version 24.0; Chicago, IL, USA). A P<0.05 (two-tailed) was considered significant for all tests.

## Supplementary Material

Supplementary Figures
