# Supplementary material for: Neuritin-overexpressing transgenic mice demonstrate enhanced neuroregeneration capacity and improved spatial learning and memory recovery after ischemia-reperfusion injury
Source: Aging (Albany NY). 2020 Dec 15;13(2):2681–99. doi: 10.18632/aging.202318 (PMC7880330; doi:10.18632/aging.202318)
Supplement: Supplementary Figures [file aging-13-202318-s001.pdf]

SUPPLEMENTARY FIGURES

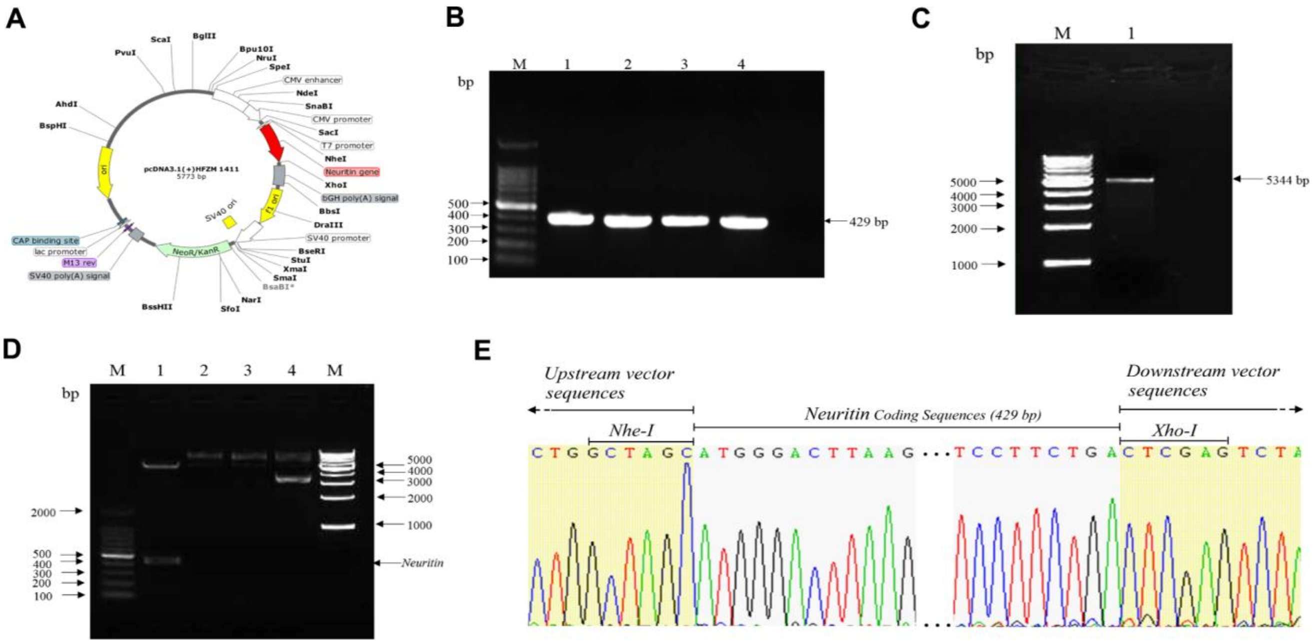

Supplementary Figure 1. Strategy for transgenic recombinant vector construction and validation. (**A**) Frame map of the pcDNA3.1(+)-neuritin recombinant vector. (**B**) Identification of purified rat Neuritin DNA by agarose gel electrophoresis. M, 100 bp DNA ladder; lanes 1-4, Neuritin DNA products digested with *Nhe-I* and *Xho-I*; lane 5, negative control. (**C**) Identification of linear pcDNA3.1(+) plasmid DNA by agarose gel electrophoresis. M, 1Kb DNA ladder; lane 1, pcDNA3.1(+) plasmid product digested with *Nhe-I* and *Xho-I*. (**D**) Identification of recombinant plasmid by restriction enzyme digestion method. M(left), 100 bp DNA ladder; M(right), 1Kb DNA ladder; lane 1, *Nhe-I*/ *Xho-I* double digestion; lane 2, *Nhe-I* single digestion; lane 3, *Xho-I* single digestion; lane 4, recombinant plasmid DNA control. (**E**) Sequencing validation of recombinant plasmid of pcDNA3.1(+)-Nrn1.

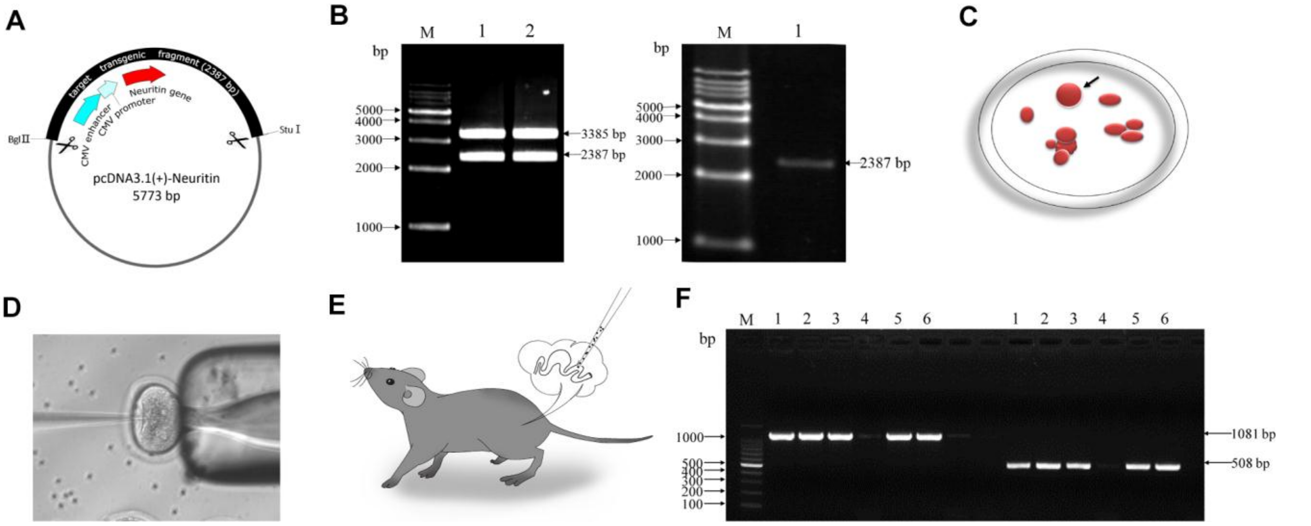

Supplementary Figure 2. Flow chart of transgenic mouse generation. (**A**) Diagram of enzyme digestion sites for vector linearization. (**B**) Agarose gel electrophoresis for the separation of transgenic DNA fragments. Left gel shows linear DNA fragments of pcDNA3.1(+) plasmid cut by *Bgl II* and *Stu I*, and right gel shows the purification of the transgenic fragment. Lane M: 1kb DNA ladder. (**C**) Collecting one cell-stage zygotes for DNA injection. (**D**) Microinjection of transgenic DNA fragments into the male pronucleus. (**E**) Transfer of injected zygotes into the oviduct of pseudopregnant mice. (**F**) PCR screening for positive transgenic offspring using specific primers (see Materials and Methods, Section 2.3).

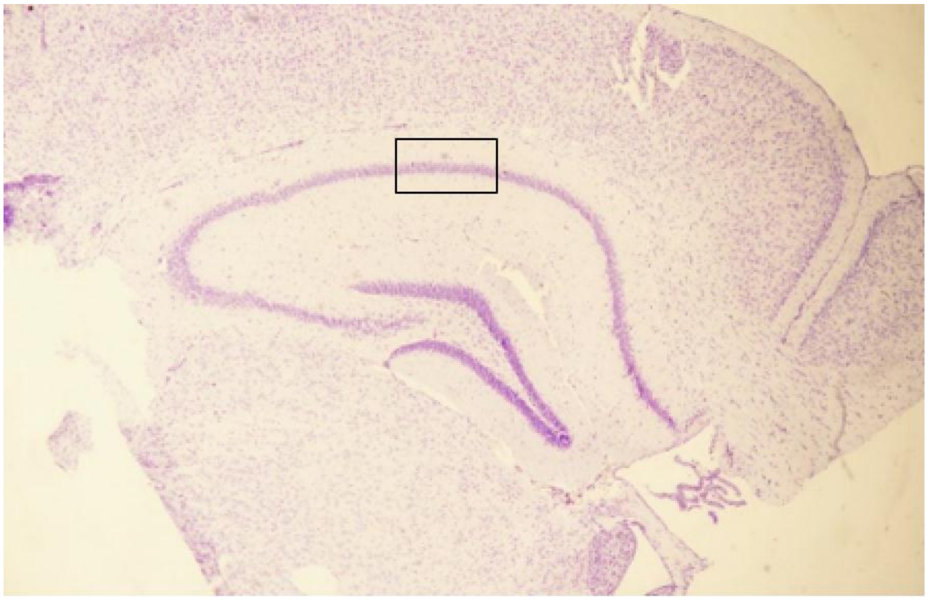

Supplementary Figure 3. Hippocampal CA1 region demarcated by the black rectangular box.
